# Insights adjusting for non-adherence in randomized clinical trials: a reanalysis of an adjuvant trial of tamoxifen duration in early breast cancer

**DOI:** 10.1038/s41416-023-02420-w

**Published:** 2023-09-11

**Authors:** Fabiola Giudici, Barbara Pistilli, Ines Vaz-Luis, Maryam Karimi, Suzette Delaloge, Thomas Bachelot, Stefan Michiels, Aurelie Bardet

**Affiliations:** 1 Bureau de Biostatistique et d’Epidémiologie, Gustave Roussy, Université Paris-Saclay, Villejuif, France; 2 Oncostat U1018, Inserm, Université Paris-Saclay, Equipe labellisée Ligue Contre le Cancer, 114 Rue Edouard Vaillant, Villejuif, France; 3 Department of Cancer Medicine, Gustave Roussy, 114 Rue Edouard Vaillant, Villejuif, France; 4 Breast Cancer Survivorship Group, INSERM Unit 981, Gustave Roussy Institute, 114 Rue Edouard Vaillant, Villejuif, France; 5 Department of Medical Oncology, Centre Léon Bérard, 28 Rue Laënnec, Lyon, France

**Keywords:** Statistics, Breast cancer

## Abstract

**Background:**

Several randomized clinical trials provide evidence of the survival benefit of extended adjuvant tamoxifen in women with estrogen receptor (ER)-positive early breast cancer (BC). However, non-adherence may lead to underestimate treatment effects using intention to treat (ITT) methods. We reanalyzed a randomized trial using contemporary statistical methods adjusting for non-adherence.

**Methods:**

The TAM01 study was a phase 3 trial including women with early BC, who had completed 2–3 years of adjuvant tamoxifen between 1986 and 1995. Participants were randomly assigned to continue tamoxifen up to 10 years or to discontinue the treatment at randomization. Invasive disease-free survival (iDFS) and overall survival (OS) were estimated using marginal structural models (MSM) and rank preserving structural failure time model (RPSFTM).

**Results:**

Of 3830 patients enrolled, 2485 were randomized to extended tamoxifen, and 1345 to treatment discontinuation. The 10-year non-adherence rate in the extended group was 27.2%. Among women with ER-positive BC (*n* = 2402), extended tamoxifen was associated with a 45% and 21% relative improvement in iDFS by MSM and RPSFTM, respectively (Hazard Ratio (HR), 0.55; 95% Confidence Interval (CI), 0.48–0.64 and HR, 0.79; 95%CI, 0.67–0.95, respectively), a considerable greater benefit than in the ITT analysis (HR, 0.90; 95%CI, 0.81–0.99). The OS reanalysis revealed a substantial benefit of extended tamoxifen (MSM: HR, 0.70; 95%CI, 0.59–0.83; RPSFTM: HR, 0.85; 95%CI, 0.67–1.04), compared to the ITT analyses (HR, 0.94; 95%CI, 0.84–1.07).

**Conclusion:**

This analysis emphasizes both the importance of adherence to hormonotherapy in hormone-receptor positive early BC and the usefulness of more complex statistical analyses.

## Introduction

Despite meaningful incremental improvements in screening, local treatment and adjuvant therapies, including endocrine therapies (AETs), estrogen receptor (ER)-positive breast cancer (BC) remains associated with a significant long-term risk of late relapse. Recommendations for extended AETs after 5 years are evolving due to recently published trials results: in patients at intermediate and high risk of recurrence, current guidelines recommend 7 to 10 years of adjuvant endocrine therapy, including at least 5 years of aromatase inhibitors (AI) given either upfront or as part of an early switch strategy after 2–3 years of tamoxifen [1]. Recently, the final results of the GIM4 phase 3 trial [2] showed that at a median follow-up of more than 12 years, disease-free survival (DFS) and overall survival (OS) outcomes were significantly improved in postmenopausal patients with ER-positive BC who, after adjuvant tamoxifen for 2–3 years, received 5 years of letrozole extension compared to the standard 2–3 years of letrozole (hazard ratios (HRs), 0.78; 95% confidence interval (CI), 0.65–0.93 and 0.77; 0·60–0.98, respectively). Safety and tolerability issues may hinder adherence to chronic treatments, however, leading to either switching treatment to another endocrine therapy, or discontinuation, according to the clinician’s or patient’s decision. Cross-sectional, retrospective, and longitudinal studies estimated adherence to treatment as averaging 79% in the first year, decreasing to 56% in the fourth and fifth year [3]). Several studies [2, 4–6] showed that adherence to tamoxifen is often suboptimal, ranging from 53% to 86%, and that its proportion decreases over time. The factors involved in non-adherence to tamoxifen treatment can be patient-related, therapy-related (duration, side effects) or associated with health care system factors such as an unsatisfactory patient–health care provider relationship [7].

Suboptimal adherence to endocrine therapy can affect BC outcomes as soon as the patients become non-adherent. We previously showed that biochemical non-adherence to tamoxifen over the first year was associated with an absolute 5.9% increase in the risk of distant recurrences at 3 years [8] (HR, 2.31; 1.05-5.06; *p* = 0.036). Moreover, discontinuation of AET is associated with increased all-cause mortality [9] (HR, 1.26; 1.09–1.46), and related to reduced DFS [10] (HR, 1.45; 1.09–1.93).

Although the prognostic relevance of adherence is well established, survival outcomes in most trials are analyzed according to the intention-to-treat (ITT) principle, which means that all patients who were enrolled in the trial are included in the analyses and are analyzed according to their randomized treatment assignment. Where there is considerable non-adherence to assigned treatment and late survival events, a frequent situation in trials with long-term follow-up, the on-treatment effect may be underestimated [11]. Simple methods of adjustment for treatment switch have been used historically in health technology assessments, which are based on the exclusion of switchers from the analysis or censorship of data at the time of their switch. These methods can create bias in treatment effect assessment, because treatment switch can be related to prognosis [12]. Recent recommendations indicate that these simple approaches should be avoided in the estimation of survival outcomes and replaced with advanced statistical techniques that are either randomization- or observational-based, allowing a robust theoretical treatment effect to be estimated in the absence of non-adherence [13–16].

These recent techniques are structural methods, relying on counterfactual survival times (i.e. survival times that would have been observed in the absence of non-adherence [17, 18]). Adjusting for non-adherence thus appears to be crucial in order to provide reliable evidence of a treatment effect in an adherent population, and to drive evidence-based medical decisions. Our aim was to apply these methods (inverse probability of censoring weights (IPCW), marginal structural models (MSM) and rank preserving structural failure time model (RPSFTM)) to a previously published large scale randomized phase 3 trial regarding the duration of tamoxifen (TAM01 study [19]) in order to provide robust and reliable estimates of extended adjuvant tamoxifen treatment benefit on survival outcomes in early BC when patients actually take the drug as assigned over the whole treatment period.

## Methods

### TAM01 study [19]: design and data derivation

The TAM01 trial is a randomized multicenter open-label superiority phase 3 trial that assessed the effects of extended tamoxifen treatment on recurrence and mortality in early BC, the design and conduct of which have been described elsewhere [19]. Briefly, the double parallel design included 3793 women in 20 cancer centers (mainly in France), enrolled between September 1986 and May 1995, aged up to 75 years, with early BC without evidence of local-regional or distant recurrence, and 2 to 3 years of tamoxifen exposure at randomization: patients were randomly allocated to the short term (ST) arm (tamoxifen was to be stopped immediately after randomization) or to the long term (LT) arm (patients continued tamoxifen for a further 10 years). Daily doses of tamoxifen ranged from 20 to 40 mg. The adherence to treatment was assessed at the patients’ follow-up visits based on patient declaration and recorded by the case report forms. The protocol was approved by the Caen Committee for the Protection of Persons in Biomedical Research.

TAM01 trial’s primary endpoint was DFS while OS was a secondary. DFS was defined as the time elapsed between inclusion and local or regional recurrence, distant metastases or death from any cause. Patients who did not experience any recurrence, metastases or died were censored at date of last news. OS was defined as the time elapsed between inclusion and death, regardless of its cause. Patients who were still alive (including those lost to follow-up) were censored at the last known date they were alive. In the original trial publication [19], an ITT analysis found that the LT arm was associated with a significantly better DFS compared to the ST arm (7-year DFS were 78% and 72%, respectively), but no substantial OS advantage was noted (7-years OS was 79% in both groups). The current analyses consider actual adherence to the assigned arm: adherence was reported by patients through scheduled hospital visits.

Patients from the LT arm and patients from the ST arm, with prolonged exposure after treatment, were therefore, allocated according to true tamoxifen exposure to a cohort of “12 years tamoxifen (extended group)”, while patients from the ST arm or LT arm who discontinued at randomization were included in a cohort of “2–3 years tamoxifen (short-term group)”.

The revised definition of invasive DFS (iDFS) reported by DATECAN initiative [20] has been considered as regards endpoints: invasive/in situ contra lateral BC and second primary invasive cancer were included in the composite event. The survival data (for OS) were updated in September 2015.

#### Statistical analysis

Statistical analyses were first performed on the ITT population, which included all randomized patients. The endpoints in this post-hoc analysis were iDFS and OS: HRs and associated 95% CIs were estimated using an unadjusted Cox proportional hazards model. We used the Schoenfeld test to check the proportionality assumption. Median follow-up was estimated with a reverse Kaplan-Meier estimator [21].The presence of tamoxifen discontinuation in the “treated patients” cannot be considered random and can create a bias in treatment effect estimation if not properly accounted for. The purpose of causal inference methods when treatment is stopped relies on the construction of counterfactual patients (“pseudo-population”), in order to mimic the conditions of perfect randomization throughout the trial duration. These methods, called “structural methods”, allow to estimate the true benefit of the experimental drug on survival endpoints that would have been estimated if there were no early tamoxifen discontinuation. There are several methods. Considering the specificities of the TAM01 trial (average proportion of switchers, variety of prognostic data collected and large sample size [22]), the IPCW, MSM, and RPSFTM were used to adjust survival estimates for non-adherence to treatment. Table 1 summarizes models characteristics. The following baseline covariates were used in order to calculate the probability of treatment adherence in the complete MSM: age, year of initial treatment, surgery (yes/no), nodal status (positive/negative), estrogen receptor status (positive/negative), radiotherapy (yes/no), chemotherapy (yes/no) and dose of tamoxifen (≤20 mg/>20 mg). Several sensitivity analyses were performed to assess the robustness of the results, either regarding methodological implementation of methods (optimal discretization of time for MSM, recensoring of follow-up and exploration of the underlying assumption of RPSFTM) or clinical questions (reallocation of patients between arms, exploratory subgroups analyses). Details are reported in Supplementary Methods 1.

**Table 1:** Methodology: Summary description and key assumptions of selected statistical methods to adjust for non-adherence to assigned treatment.

Advanced Model	Summary Description	Key assumptions
Inverse Probability of Censoring Weighting (IPCW)	**Main principle:** The IPCW method uses patient data to create an artificial analysis set of fully-adherent patients. To adjust for treatment discontinuation, remaining patients who continued the treatment but have similar characteristics are reweighted according to the inverse probability of non-stop of treatment. Steps - calculation of probability of treatment adherence using baseline covariates that predict both treatment discontinuation and outcomes (iDFS and OS) - a ‘pseudo-population’ is created using the adherence weights - treatment effects were estimated with a weighted Cox proportional hazards model to calculate hazard ratios (HRs) and 95 % confidence intervals (CIs).	**No unmeasured confounders**: all baseline covariates and time-dependent confounders that predict stop of treatment and survival outcomes are included.
Marginal Structural Model (MSM)	**Main principle**: combination of IPCW and weighting for follow-up censorship MSM recreates the population that would be seen without dropouts and without stopping treatment Steps - calculation of the probability of treatment adherence and follow-up using baseline covariates that predict treatment stop, drop-out and outcomes (iDFS and OS) - a ‘pseudo-population’ is created using the final weights defined by multiplying the adherence weights and the censoring weights - treatment effects were estimated with a weighted Cox proportional hazards model to calculate HRs and associated 95% CIs	**No unmeasured confounders**: all baseline covariates and time-dependent confounders that predict stop of treatment and survival outcomes, and drop-out are included.
Rank Preserving Structural Failure Time Model (RPSFTM)	Main principle: estimation of treatment effect using grid-search process, based on CTE assumption Steps: – survival is defined in treatment group according to periods of treatment (with treatment effect ψ) and periods of non-treatment (without any treatment effect) – ψ is estimated through g-estimation, and the treatment effect for fully-adherent patients is derived Treatment effects were estimated with a Cox proportional hazards model to calculate HRs.	**Perfect randomization assumption**: the two groups are comparable beyond chance and if they had both received control treatment then their survival would be the same on average -**Common treatment effect assumption**: experimental treatment effect is the same regardless of when it is given (in this case, the no-treatment effect is the same at randomization or after tamoxifen discontinuation)

*CTE* common treatment effect.

#### Missing data

Some clinical variables were incomplete in the original TAM01 trial. ER receptor status in particular had a considerable amount of missing information. It cannot be ensured that data are missing completely at random [23], and so two approaches were considered for ER status and clinical/demographic variables with sporadic missingness in order to consider missingness at random (Supplementary Results: Table S1 and Fig. S1). In the first approach, presented as the main result in this study, missing data were imputed using multiple imputation using the chained equations (MICE) technique [24]. Details about missingness by variable and implementation of MICE [25, 26] are reported in Supplementary Methods 2. The second simple approach consists of considering missing data as a specific category of the variable (analysis reported in Supplementary Results).

All statistical analyses were performed using R software version 4.0.2 (2020) and StataCorp LP. Stata statistical software (14.2). The statistical packages used for all analysis analyses performed are reported in Supplementary Methods 3.

## Results

### Population and tamoxifen adherence

**Fig. 1:**
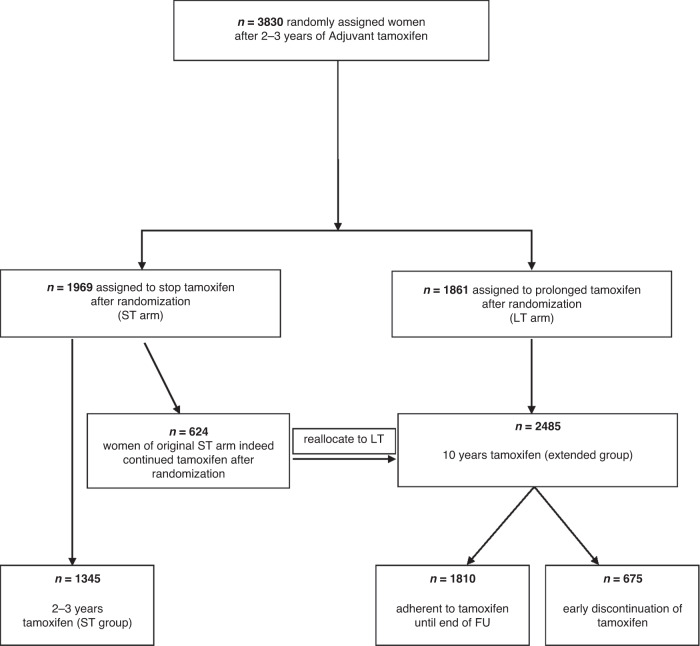
Study flow chart. Diagram of the study reporting randomization and reallocation of patients enrolled in the trial according to tamoxifen treatment.

Figure 1 shows patient allocation in the reanalysis. Of 3830 patients enrolled, 2485 actually received extended tamoxifen, and only 1345 stopped the treatment. Table 2 shows the characteristics of the two cohorts: “2–3 years tamoxifen (ST group)” and “10 years tamoxifen (extended group)” patients. Although the newly defined groups do not correspond to randomization arms, the clinico-pathological parameters are well balanced between groups. The median duration of follow-up was 8.1 (interquartile range (IQR): 4.7–11.0) years for iDFS and 10.4 (IQR: 8.0–12.8) years for OS.

**Table 2:** Clinical and pathological characteristics of enrolled patients.

	Groups	
	2–3 years tamoxifen (ST group) *n* = 1345	10 year tamoxifen (extended group) *n* = 2485
Age (years)		
Median (IQR)	63 (57–68)	62 (57–68)
Years of initial treatment		
<1990	848 (63.0%)	1614 (64.9%)
≥1990	496 (36.9%)	863 (34.7%)
Unknown	1 (0.07%)	8 (0.3%)
Type of breast surgery		
No surgery	48 (3.6 %)	78 (3.1%)
Lumpectomy	579 (43.0%)	1265 (50.9%)
Mastectomy	715 (53.2%)	1130 (45.5%)
Unknown	3 (0.2%)	12 (0.5%)
Tumor size		
pT0	31 (2.3%)	102 (4.1%)
pT1	288 (21.4%)	633 (25.5%)
pT2	719 (53.5%)	1209 (48.7%)
pT3-4	204 (15.2%)	352 (14.2%)
Unknown	103 (7.7%)	189 (7.6%)
Nodal status		
Negative	381 (28.3%)	846 (34.0%)
Positive	961(71.5%)	1634 (65.8%)
Unknown	3 (0.2%)	5 (0.2 %)
Estrogen receptor status		
Positive	806 (59.9%)	1596 (64.2%)
Negative	180 (13.4%)	248 (10.0%)
Unknown	359 (26.7%)	641 (25.8%)
Tamoxifen dosage		
≤20 mg	427 (31.8%)	1355 (54.5%)
> 20 mg	876 (65.1%)	1108 (44.6%)
Unknown	42 (3.1%)	22 (0.9%)
Adjuvant chemotherapy		
No	956 (71.1%)	1674 (67.4%)
Yes	384 (28.6%)	785 (31.6%)
Unknown	5 (0.3%)	26 (1.0%)
Adjuvant radiotherapy		
No	184 (13.7%)	296 (11.9%)
Yes	1158 (86.1%)	2175 (87.5%)
Unknown	3 (0.2%)	14 (0.6%)

*IQR* interquartile range.

**Fig. 2:**
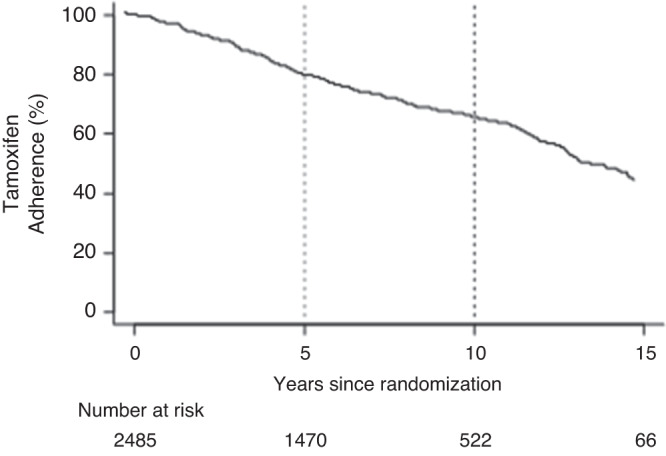
Tamoxifen adherence in women taking tamoxifen for 10 years (extended group, *n* = 2485 women). Dotted lines: 5-years and 10-years tamoxifen adherence.

Of the 2485 patients in the extended group, 675 (27.2%) stopped tamoxifen intake during the follow-up. Figure 2 shows the prevalence of declared use of tamoxifen over time (since randomization) in this population. Treatment adherence decreased at a constant rate over time: overall, 82.6% and 62.9% of patients were still under therapy, respectively, at 5 and 10 years from randomization.

### Survival analysis

#### iDFS outcome

The ITT unadjusted analysis for the whole cohort, and among women with ER positive BC, showed that iDFS was slightly improved for patients assigned to 12 years tamoxifen compared to those assigned to 2–3 years (HR = 0.90 (95% CI: 0.81–0.99), *p* = 0.045 and HR = 0.90 (0.79–1.03), *p* = 0.118, respectively).

**Fig. 3:**
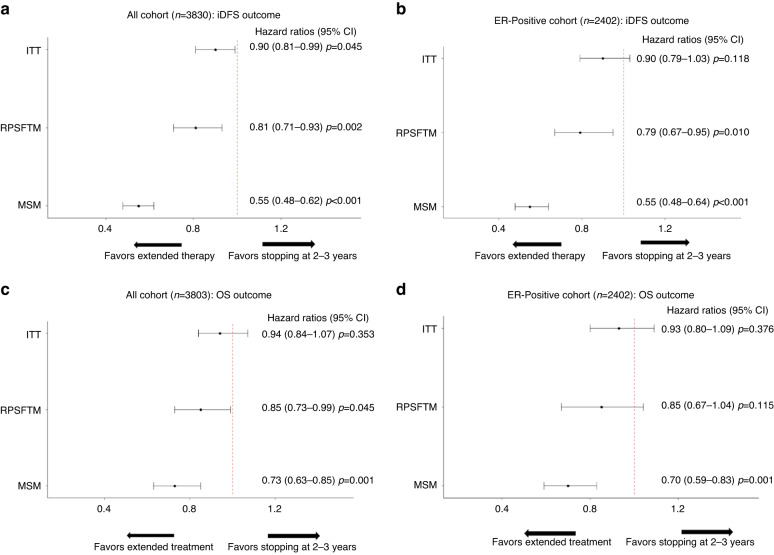
Forest plots showing ITT, RPSFTM and MSM estimates of tamoxifen treatment effect on iDFS and OS. Forest plots of hazards ratios for iDFS in all cohort (**A**), in estrogen positive breast cancers (**B**) and for OS in all cohort (**C**) and in estrogen positive breast cancers (**D**). Hazard ratios were calculated by comparing the extended tamoxifen group (12 years) and the short-term group (2–3 years) for all methods (intention to treat, marginal structural models and rank preserving structural failure time models). ITT intention-to-treat, iDFS invasive disease-free survival, OS overall survival, MSM marginal structural models, RPSFTM Rank Preserving Structural Failure Time Model; CI confidence interval, p *p* value.

The results of the adherence-adjusted analyses are presented in Fig. 3A, B. All causal inference methods suggest a stronger benefit of prolonged tamoxifen on iDFS: in ER-positive women, the HRs using MSM and RPSFT modeling were 0.55 (0.48–0.64) and 0.79 (0.67–0.95), respectively (*p* < 0.001 for MSM and *p* = 0.010 for RPSFTM).

#### OS outcome

compared to the original TAM01 trial, and according to the ITT principle, the OS endpoint did not achieve statistical significance. When implementing an unadjusted Cox model, the relative hazards comparing the extended tamoxifen group and the ST group were 0.94 (0.84–1.07) in all cohort and 0.93(0.80–1.09) in ER positive women. The counterfactual statistical methods revealed that the observed survival benefit was likely to be underestimated (Fig. 3C, D): HRs were statistically significant across structural methods: in the whole cohort the estimates of the HRs given by MSM and RPSFTM were 0.73 (0.63–0.85) and 0.85 (0.73–0.99), respectively. In women with ER positive BC, HRs ranged from 0.70 (0.59–0.83) to 0.85 (0.64–1.04) using the MSM and RPSFT models, respectively.

### Sensitivity analyses

Several sensitivity analyses were conducted in the whole cohort in order to evaluate the robustness of the counterfactual methods. In the MSM model, estimate accuracy increases as interval length decreases: the HRs which calculate weights using 180 days as the time-interval (compared to 50 days selected for the main analysis) for the extended group compared to the ST group, were 0.57 (0.51–0.65) and 0.77 (0.66–0.89), evaluating iDFS and OS outcomes, respectively. The RPSFTM method produced an iDFS HR of 0.72 (0.59–0.88) and 0.81 (0.71–0.93) and an OS HR of 0.76 (0.58–0.99) and 0.85 (0.73-99) with and without recensoring, respectively (Supplementary Table S2, Fig. S2). The investigation of survival outcomes in ER negative patients (*n* = 428) confirmed a stronger treatment effect from tamoxifen in the ER positive extended cohort: in terms of OS the analysis performed for ER-negative patients showed no differences between the ST and 12-year tamoxifen groups (HR = 0.74 (0.50–1.11), *p* = 0.144 and HR = 0.78 (0.51–1.18), *p* = 0.233, applying the MSM and RPSFTM methods, respectively). Sensitivity analyses results are reported in Supplementary Results (Supplementary Table S2).

## Discussion

Tamoxifen is a common and established adjuvant therapy, particularly for premenopausal patients with ER-positive BC [27]. Traditionally, treatment has been given for around 5 years, but many women remain at risk of relapse for 10 years or more.

In this reanalysis of a large randomized trial of women with ER-positive BC, at a median follow-up of more than 8 years, and after adjustment for treatment adherence, the patients in the extended therapy group (12 years) had a significantly improved iDFS and OS than those who received ST therapy (2–3 years) (iDFS HR of 0.55 (0.48–0.64) and of 0.79 (0.67–0.95); OS HR of 0.70 (0.59–0.83) and of 0.85 (0.64–1.04) using MSMs and RPSFTM, respectively). These estimates indicated a significantly larger treatment effect with respect to ITT analysis.

This analysis is still significantly relevant, provided that adjuvant endocrine therapy for 5 years or longer remains a cornerstone in the treatment of patients with HR + BC.

The Adjuvant Tamoxifen, To Offer More? (aTTom [28]) and Adjuvant Tamoxifen: Longer against Shorter (ATLAS [29]) studies are the most recent trials to investigate the question of extended tamoxifen treatment (5 versus 10 years) without any other AIs. Results show a time-dependent reduction in the risk of recurrence and death, with a beneficial effect that was small during treatment uptake and started to increase from year 10 onwards, and emerging in the second decade, after treatment discontinuation. Survival outcomes were investigated in both trials with ITT analysis, although adherence to extended tamoxifen therapy dropped to 75–80% as early as 2–3 years after randomization.

To our knowledge, the treatment benefits for survival have not been estimated in any of the previous published studies investigating extended tamoxifen, adjusting for adherence. Colleoni et al. [30] analyzed the BIG 1–98 breast trial data using the IPCW modeling method, but for the issue of crossover treatment. The authors also implemented IPCW only, and no other causal inference methods.

Some important issues need to be addressed: nowadays, tamoxifen 20 mg/day for 5 years is still considered the standard of care to reduce the risk of recurrence/death in premenopausal women with ER-positive BC who are at low risk of recurrence [31]. In the TAM01 study, only half of patients received the current standard dose of tamoxifen. Patient characteristics and treatment dosing are related to the enrollment period: in the late 1980s and early 1990s, tamoxifen was widely used in treating postmenopausal receptor-negative BC [32] and women could receive tamoxifen dose of 40 mg/day [33]. Similarly, it is common in old trials investigating adjuvant tamoxifen treatment effect, to include women with unknown receptor status [27]. TAM01 was well performed in its historical context and our aim is not to adapt it to current treatment guidelines but apply of statistical methods that properly estimate tamoxifen treatment effect in the presence of non-adherence.

In future research, we plan to complement the current analysis using the contemporary CANTO cohort [34], which includes a fine-tuned data collection of prognostic factors for treatment adherence, management of adverse effects and quality of life. Finally, although adjustment methods such as MSM and RPSFTM are likely to produce less bias than naive per‐protocol adjustments, it is important to assess the validity of underlying assumptions [35]. The most demanding assumption for MSM is the absence of unmeasured confounders in the treatment allocation and switching process. Even if several potential confounders have been considered, it cannot be ensured that all confounders are known or measured, especially as time-dependent covariates were not available. The absence of high level of outliers in the weight estimation, however, works in favor of the absence of unmeasured confounding. The RPSFTM method relies on the untestable assumption that common treatment effect (CTE), that is treatment effect vs non-treatment, is the same whatever the time of treatment switch. This hypothesis is somehow related to the reasons for switching (a patient switching after progression probably received less benefit from the treatment), which are unknown in this study. Sensitivity analyses do not provide strong elements for the invalidity of the CTE hypothesis, however. The variable effect size obtained using MSM and RPSFTM for the iDFS outcome, with a higher estimated treatment effect for MSM, might be related to the different underlying assumptions. The MSM method may be more appropriate in trials with a relatively large sample size, in which non-adherence is observed in a moderate proportion of patients, and sufficient information regarding potential confounding factors is available [36]. The RPSFTM would be preferable for smaller trials with relatively little information on covariates and is also suitable for trials in which massive non-adherence is observed [37]. The published data of several cancer trials [30, 37–39] recently showed that using the adherence-adjusted methods resulted in the experimental treatment having a less biased effect on OS than reported in the ITT analysis. However, causal methods are still rarely used in clinical trials settings [40]: there could be several reasons for this. First, they require data transformations and modeling steps, each including methodological choices, that may have limited their easy use and can be computationally intensive. Second, assumptions underpinning causal methods are untestable. Third, the counterfactual approach is more difficult to explain than the more common ITT approach. Notwithstanding, Latimer and al., using a simulation study [41] concluded that the MSM and RPSFTM methods consistently produced less bias than ITT analysis.

## Conclusions

Conventional ITT analysis is a valid and universally accepted analytic approach to test two treatment strategies, but it does not control for potential biases due to treatment discontinuation. Additional analyses should be considered in long-term trials with substantial non-adherence to randomized treatment, so as to assess the effect of non-adherence on the estimate from the ITT analysis. In the context of the TAM01 trial, adjusting for treatment adherence using the MSM and RPSFT methods, reveals that long-term tamoxifen has a greater protective effect on iDFS and OS in ER-positive early BC patients. These structural methods emphasize the benefit of adhering to hormonotherapy in hormone-receptor positive early BC.

### Supplementary information

Supplementary Material

## Data Availability

The data that support the findings of this study can be requested from the corresponding author upon requests that comply with the local data protection regulations.

## Code availability

The R code for the analysis used in this article is available upon request.
