# Supplementary material for: Insights adjusting for non-adherence in randomized clinical trials: a reanalysis of an adjuvant trial of tamoxifen duration in early breast cancer
Source: Br J Cancer. 2023 Sep 11;129(9):1516–23. doi: 10.1038/s41416-023-02420-w (PMC10628101; doi:10.1038/s41416-023-02420-w)
Supplement: Supplementary file 1 — Supplementary Material [file 41416_2023_2420_MOESM1_ESM.docx]

**INSIGHTS ADJUSTING FOR NON-ADHERENCE IN RANDOMIZED CLINICAL TRIALS: A REANALYSIS OF AN ADJUVANT TRIAL OF TAMOXIFEN DURATION IN EARLY BREAST CANCER**

**Fabiola Giudici^1,2^, PhD ; Barbara Pistilli^3^, MD ; Ines Vaz-Luis^3,4^, MD; Maryam Karimi^1,2^, PhD; Suzette Delaloge^3^, MD; Thomas Bachelot^5^, MD; Stefan Michiels^1,2^, PhD; Aurelie Bardet^1,2^,MSc**

^1^Bureau de Biostatistique et d'Epidémiologie, Gustave Roussy, Université Paris-Saclay, Villejuif, France

^2^Oncostat U1018, Inserm, Université Paris-Saclay, Equipe labellisée Ligue Contre le Cancer, Villejuif, France

^3^Department of cancer Medicine, Gustave Roussy, Villejuif, France.

^4^Breast Cancer Survivorship Group, INSERM Unit 981, Gustave Roussy Institute, Villejuif, France.

^6^Department of Medical Oncology, Centre Léon Bérard, Lyon, France.

**Supplementary Material**

TABLE OF CONTENTS

[**Supplementary Methods 1** 2](#_Toc126039037)

[**Statistical Analysis** 2](#_Toc126039038)

[**Sensitivity analysis to evaluate the robustness of MSM and RPSFTM estimates** 2](#_Toc126039039)

[**Supplementary Methods 2** 2](#_Toc126039040)

[**Statistical Analysis: Missing Data** 2](#_Toc126039041)

[**Multiple imputation** 2](#_Toc126039042)

[**Supplementary Methods 3** 3](#_Toc126039043)

[**Statistical Analysis: Statistical Packages** 3](#_Toc126039044)

[**IPCW-MSMs:** 3](#_Toc126039045)

[**RPSFTM:** 3](#_Toc126039046)

[**Supplementary Results:** 4](#_Toc126039047)

[**Table S1: Distribution of Estrogen Receptor status across clinico-pathological variables** 4](#_Toc126039048)

[**Table S2: Sensitivity analyses to evaluate the robustness of results from structural methods** 5](#_Toc126039049)

[**Figure S1: Aggregation plot of the variables used for multiple imputation of ER status.** 6](#_Toc126039050)

[**Figure S1: Comparison of counterfactual survival times in RPSFTM** 6](#_Toc126039051)

[**Supplementary References** 7](#_Toc126039052)

# **Supplementary Methods 1**

## **Statistical Analysis**

## **Sensitivity analysis to evaluate the robustness of MSM and RPSFTM estimates**

**MSM*:*** investigation of the impact of the width of time intervals on weights computation. The results were detailed in **Supplementary Results (Supplementary Table S2).**

**RPSFTM**: -estimation of HR were computed with and without recensoring survival times to avoid informative censoring bias (**Supplementary Figure S2).** In a long follow-up trial, recensoring was associated with a substantial loss of longer-term survival information with an overestimation of treatment effect respect to no-recensoring HR estimate. In the main analysis, HR without recensoring is reported, according to Latimer at al. guidelines^1^, which specify that re-censoring should not always represent the default approach when the objective is to estimate long-term survival times.

**-**verification of the CTE hypothesis given the fact that the reasons for treatment discontinuation were unknown, two hypothetical opposite scenarios were simulated. In more details, tamoxifen efficacy for the non-adherent extended cohort is decreased by 20% (for example, this could be the case if treatment discontinuation is due to progression), and treatment effect for extended cohort who early discontinued tamoxifen is increased by 20%.

The results were detailed in **Supplementary Results (Supplementary Table S2).**

**Sensitivity analysis performed for both methods:**

Evaluation of the impact on HR’s estimate due the exclusion of the 624 women in the original ST arm who continued the tamoxifen intake after randomization (**Supplementary Table S2**)

# **Supplementary Methods 2**

## **Statistical Analysis: Missing Data**

## **Multiple imputation**

ER receptor status had a considerable amount of missing information. Overall, 1000 (26%) women had a missing ER status. In addition, some degree of missing data was present in other clinical variables. For this reason, we used a multiple imputation by chained equations (MICE) technique^2^ to impute ER status and clinical/demographic variables with sporadic missingness. Briefly, the main idea of imputation technique is based on the following concept: modeling of each variable with missing observations conditional on all other variables (including those with missing values) and stochastic imputation of the missing observations based on the obtained conditional distributions. With a sporadic missingness pattern as is present in the trial data, the imputation procedure cycles through these conditional models to produce a specified number of imputed datasets. Thus, the final imputed dataset would contain not only imputed ER status but also other variables that initially had missing information (e.g. dose of tamoxifen, node, etc.). **Supplementary Figure S1** lists variables used for multiple imputation of missing ER status, along with the percentage of missing data for each variable. These variables were chosen based on clinical importance associated with ER status. We imputed missing ER status under the missing at random (MAR) assumption^3^, which is required by the MICE technique. The MAR assumption is not testable; however, we assessed association of known and unknown ER status with clinical variables used in the imputation model (**Supplementary Table S1**). Missing ER status appeared to be associated with several of these variables, thus providing plausibility for the MAR assumption after conditioning on these associated variables. We used *mice* R-package^4^ and generated 20 imputed datasets for analyses (this number is required to reduce sampling variability from the imputation process^5^). Finally, we analyzed each imputed dataset separately, and then HRs estimates (using MSM and RPSFTM methods) were combined using Rubin's rules^6^.

# **Supplementary Methods 3**

## **Statistical Analysis: Statistical Packages**

## **IPCW-MSMs:**

-The creation of data panel (i.e. split follow-up period in time intervals with matching patient status and covariates) was made using SurvSplit function of *survival* package^7^ of R statistical software^8^ (version 4.0.2).

- The computation of IPCW-MSM weights (via logistic regression model) was performed using iptwm function in the *ipw* package^9^. This package let to check graphically the weight distribution with ipwplot function used to assess the IPCW from adequately formatted data. iptwm is programmed for situation of crossover from control arm to experimental arm. We adapted this function to our trial in which patients in the experimental arm stopped tamoxifen treatment and there is no switching from control to experimental: for 2-3 years tamoxifen (controls), the time-varying weight were set to one throughout the follow-up. Similar results were obtained using a recent R-package^10^ (*ipcwswitch*) suitable even for early discontinuation of treatment but more computationally expensive because it has been created in particular to compute weights in the presence of time-dependent covariates (in TAM01trial we considered only baseline covariates)

-The resulting weights were applied in the analysis of iDFS and OS via weighted Cox regression model (*survival* package of R)

## **RPSFTM:**

For the RPSFTM, counterfactual survival times were calculated assuming an ever-treated structural model with a long-rank test using interval bisection employed for g- estimation. These calculations were performed using the Stata module^11^ strbee that is appropriate also to consider situation in which the switching event happens in the experimental arm (such as in TAM01 trial). -hr option estimates the hazard ratio (with a 95% test-based confidence interval^12^) comparing experimental arm if always treated with control arm never treated.

-kmgraph option displays counterfactual survival times assuming both randomized groups received only the control treatment (in our case no tamoxifen intake). - psimult(k) allows the acceleration factor to vary between individuals: this option is typically used to assess sensitivity to departures from the CTE assumption. In Stata this option is directly applicable for common situation in which the switching process happen from control arm to experimental arm:  for example, to fit the model with the effect of treatment 30% smaller in the control arm, it would specify psimult(k) where k is 1 in the experimental arm and 0.7 in the control arm. We adapted the command to the situation of stop of treatment hypothesing two scenarios: 20% of less tamoxifen efficacy for the treated patients that might have stopped the treatment for progression (k=0.80) and 20% of gained treatment effect for treated patients who ealy discontinued tamoxifen for other reasons respect to non-treated (k=1.20).

-We used also the package^13^ *rpsftm***,** available in R statistical software, to implement this structural model**:** differently to strbee STATA module, the rpsftm function is not ready to use for the situation of stop of treatment but it is necessary to manipulate data in order to correctly use it. Moreover, rpsftm, provide only g estimation and the acceleration failure parameter with 95% confidence interval without a direct estimate of HR.

# **Supplementary Results:**

## **Table S1: Distribution of Estrogen Receptor status across clinico-pathological variables**

|  | Estrogen receptor status | | | |
| --- | --- | --- | --- | --- |
| All Cohort | **Positive**  **(n=2402)** | **Negative**  **(n=428)** | **Unknown**  **(n=1000)** | **Total**  **(n=3830)** |
| Age (N,%) |  |  |  |  |
| <50 | 115 (62.2%) | 32 (17.3%) | 38 (20.5%) | 185 |
| >=50 | 2,287 (62.7%) | 396 (10.9%) | 962 (26.4%) | 3,645 |
|  |  |  |  |  |
| Nodal status (N,%) |  |  |  |  |
| N+ | 1,705 (65.7%) | 301 (11.6%) | 589 (22.7%) | 2,595 |
| N- | 697 (56.8%) | 124 (10.1%) | 406 (33.1%) | 1,227 |
| Unknown | 0 | 3 (37.5%) | 5 (62.5%) | 8 |
|  |  |  |  |  |
| Surgery (N,%) |  |  |  |  |
| Yes | 2,365 (64.1%) | 419 (11.4%) | 906 (24.5%) | 3,690 |
| No | 33 (26.2%) | 9 (7.1%) | 84 (66.7%) | 126 |
| Unknown | 4 (28.6%) | 0 (0.0%) | 10 (71.4%) | 14 |
|  |  |  |  |  |
| Radiotherapy (N,%) |  |  |  |  |
| Yes | 2,114 (63.4%) | 364 (10.9%) | 855 (25.7%) | 3,333 |
| No | 282 (58.8%) | 63 (13.1%) | 135 (28.1%) | 480 |
| Unknown | 6 (35.3%) | 1 (5.9%) | 10 (58.8%) | 17 |
|  |  |  |  |  |
| Chemotherapy (N,%) |  |  |  |  |
| Yes | 801 (68.5%) | 158 (13.5%) | 210 (18.0%) | 1,169 |
| No | 1,590 (60.5%) | 270 (10.3%) | 770 (29.3%) | 2,630 |
| unknown | 11 (35.5%) | 0 (0.0%) | 20 (64.5%) | 31 |
|  |  |  |  |  |
| TaMoxifen dose (N,%) |  |  |  |  |
| <=20 | 1,130 (63.5%) | 115 (6.5%) | 536 (30.1%) | 1,781 |
| >20 | 1,271 (64.1%) | 313 (15.8%) | 400 (20.2%) | 1,984 |
| Unknown | 1 (1.5%) | 0 (0.0%) | 64 (98.5%) | 65 |
|  |  |  |  |  |
| Recurrence (N,%) |  |  |  |  |
| Yes | 919 (61.7%) | 183 (12.3%) | 388 (26.0%) | 1490 |
| No | 1,483 (63.4%) | 245 (10.5%) | 612 (26.2%) | 2340 |
|  |  |  |  |  |
| Death |  |  |  |  |
| Yes | 655 (61.0%) | 146 (13.6%) | 273 (25.4 %) | 1074 |
| No | 1728 (63.3%) | 282 (10.3%) | 719 (26.4%) | 2729 |
| Unknown | 19 (70.4%) | 0 (0.0%) | 8 (29.6%) | 27 |
|  |  |  |  |  |
| Stop of treatment |  |  |  |  |
| Yes | 462 (67.8%) | 64 (9.4%) | 155 (22.8%) | 681 |
| No | 1940 (61.6%) | 364 (11.6%) | 845 (26.8%) | 3149 |
|  |  |  |  |  |

## **Table S2: Sensitivity analyses to evaluate the robustness of results from structural methods**

| Invasive Disease-Free Survival Outcome | | | | Overall Survival Outcome | | |
| --- | --- | --- | --- | --- | --- | --- |
| Model: MSM HR (95% CI) P-value | | | | **Model: MSM** **HR (95% CI) P-value** | | |
| All Cohort ^1^ | 0.55 (0.48-0.62) | | <.0001 | **All Cohort** ^1^ | 0.73 (0.63-0.85) | 0.001 |
| Sensitivity Analysis Sensitivity Analysis | | | | | | |
| *Time Intervals* | |  |  | ***Time Intervals*** |  |  |
| Intervals of 180 days | | 0.57 (0.51-0.65) | <0.001 | Intervals of 180 days | 0.77 (0.66-0.89) | 0.001 |
| *Missing data* | |  |  | ***Missing data*** |  |  |
| Exclusion missing ER status (n=2830) | | 0.52 (0.45-0.61) | <0.001 | Exclusion missing ER status (n=2830) | 0.69 (0.58-0.81) | <0.001 |
| *Population* | |  |  | ***Population*** |  |  |
| Only ER- (n=428) | | 0.55 (0.38-0.79) | 0.001 | Only ER- (n=428) | 0.74 (0.50-1.11) | 0.1440 |
| Only ER missing (n=1000) | | 0.62 (0.48-0.80) | 0.001 | Only ER missing (n=1000) | 0.86 (0.64-1.15) | 0.312 |
| Exclusion of the 624 women  treated in the control group | | 0.60 (0.52-0-69) | <0.001 | Exclusion of the 624 women  treated in the control group | 0.80 (0.67-0.93) | 0.004 |
|  | |  |  |  |  |  |
| Model: RPSFTM | **HR (95% CI)** | | **P-value** | **Model: RPSFTM** | **HR (95%CI)** | **P-value** |
| All Cohort ^1^ | | 0.81 (0.71-0.93) | 0.002 | **All Cohort ^1^** | 0.85 (0.73-0.99) | 0.045 |
| Sensitivity Analysis Sensitivity Analysis | | | | | |  |
| *Recensoring* | |  |  | ***Recensoring*** |  |  |
| With re-censoring | | 0.72 (0.59-0.88) | 0.002 | With re-censoring | 0.76 (0.58-0.99) | 0.045 |
| *CTE*^2^ | |  |  | ***CTE*** |  |  |
| K=0.80  K=1.20 | | 0.77(0.63-0.91) 0.84 (0.73-0.94) | 0.002 | **K=0.80**  **K=1.20** | 0.85 (0.71-0.99) 0.90 (0.79-0.99) | 0.045 |
| *Missing data* | |  |  | ***Missing data*** |  |  |
| Exclusion missing ER status (n=2830) | | 0.78 (0.67-0.92) | 0.002 | Exclusion missing ER status (n=2830) | 0.82 (0.68-0.98) | 0.035 |
| *Population* | |  |  | ***Population*** |  |  |
| Only ER- (n=428) | | 0.75 (0.50-1.13) | 0.171 | Only ER- (n=428) | 0.78 (0.51-1.18) | 0.233 |
| Only ER missing (n=1000) | | 0.83 (0.65-1.07) | 0.149 | Only ER missing (n=1000) | 0.94 (0.70-1.27) | 0.707 |
| Exclusion of the 624 women  treated in the control group | | 0.86 (0.74-0.98) | 0.033 | Exclusion of the 624 women  treated in the control group | 0.88 (0.76-1.03) | 0.112 |

Abbreviations: HR: hazard ratio; MSM: marginal structural model; RPSFTM: rank preserving structural failure time model; CTE: Common Treatment Effect.

For MSM: where not explicitly reported, HR were estimated using width of time intervals of 50 days

For RPFTSM: where not explicitly reported, HR were estimated without recensoring

^1^HR estimated by using ER missing status considered as a category

^2^ K=0.80. fit the model with the effect of treatment 20% smaller in the treated patients who stopped treatment; K=1.20 fit the model with the effect of treatment 20% greater in the treated patients who stopped treatment;

## **Figure S1: Aggregation plot of the variables used for multiple imputation of ER status.**

The bar plot on the *left hand side* shows the proportion of missing values in each of the selected variables.

On the *right hand side*, all existing combinations of missing and non-missing values in the observations are visualized. A gray rectangle indicates missingness in the corresponding variable; a white rectangle represents available data. The frequencies of the different combinations are represented by a small horizontal bar plot.

Proportions of Missing

0.00

0.05

0.10

0.15

0.20

0.25

ER

Dose of Tam

CT

RT

Surgery

Node

Age

Stop of Tam

Recurrence

Missing in Combinations of Variables

ER

Dose of Tam

CT

RT

Surgery

Node

Age

Stop of Tam

Recurrence

73.24%

23.89%

1.436%

0.287%

0.261%

0.183%

0.157%

0.131%

0.104%

0.078%

0.078%

0.026%

0.026%

0.026%

0.026%

0.026%

0.026%

26.1%

1.7%

0.8%

0.4%

0.4%

0.2%

0.0%

0.0%

0.0%

## **Figure S2: Comparison of counterfactual survival times in RPSFTM**

**a) without recensoring and b) with recensoring for iDFS outcome to assess the performance of the method**

**a) b)**

0.00

0.25

0.50

0.75

1.00

0

50

100

150

200

analysis time

arm = 0 untreated

arm = 1 un-treated

Counterfactual for psi=-.2045387

Kaplan-Meier survival estimate

0.00

0.25

0.50

0.75

1.00

0

50

100

150

200

analysis time

arm = 0 un-treated

arm = 1 un- treated

Counterfactual for psi=-.1595053

Kaplan-Meier survival estimate

# **Supplementary References**

1. Latimer NR, White IR, Abrams KR, et al. Causal inference for long-term survival in randomised trials with treatment switching: Should re-censoring be applied when estimating counterfactual survival times? *Stat Methods Med Res*. 2019;28(8):2475-2493. doi: 10.1177/0962280218780856.

2. White IR, Royston P, Wood AM. Multiple imputation using chained equations: Issues and guidance for practice. *Stat Med* 2011;30:377–99.

3. Hayati Rezvan P, Lee KJ, Simpson JA. The rise of multiple imputation: a review of the reporting and implementation of the method in medical research. *BMC Med Res Methodol*. 2015;15:30. doi:10.1186/s12874-015-0022-1

4.van Buuren, Stef & Groothuis-Oudshoorn, Karin, 2011. "[mice: Multivariate Imputation by Chained Equations in R](https://ideas.repec.org/a/jss/jstsof/v045i03.html)," [*Journal of Statistical Software*](https://ideas.repec.org/s/jss/jstsof.html), Foundation for Open Access Statistics, vol. 45(i03).

5. Sterne J A C, White I R, Carlin J B, et al. Multiple imputation for missing data in epidemiological and clinical research: potential and pitfalls BMJ 2009; 338 :b2393 doi:10.1136/bmj.b239

6. Little RJA, Rubin DB. Statistical analysis with missing data. New York, NY: John Wiley & Sons, Inc; 2002

7. T.M. Therneau, A Package for Survival Analysis in S. 2015; version 2.38 https://[CRAN.R-project.org/package=survival](http://cran.r-project.org/package=survival)

8. R Core Team, R: A Language and Environment for Statistical Computing, R
Foundation for Statistical Computing, Vienna, Austria, 2020 <https://www.Rproject.org/>

9. W.M. van der Wal, R.B. Geskus, ipw: an R package for inverse probability weighting, J. Stat. Softw. 43 (2011) 1–23, [doi:10.18637/jss.v043.i13](https://doi.org/10.18637/jss.v043.i13).

10. Graffeo N, Latouche A, Le Tourneau C, et al. ipcwswitch: an R package for inverse probability of censoring weighting with an application to switches in clinical trials. *Comput Biol Med*. 2019;111:103339. doi:10.1016/j.compbiomed.2019.103339

11. White IR, Walker S, Babiker A. strbee: Randomization-based efficacy estimator. *The Stata Journal*. 2002;2 (2):140-50.

12. White IR, Babiker AG, Walker S, Darbyshire JH. Randomisation-based methods for correcting for treatment changes: examples from the Concorde trial. *Statistics in Medicine*. 1999;18: 2617-2634

13. Allison A, White IR, Bond S. rpsftm: An R Package for Rank Preserving Structural Failure Time Models. *R Journal*. 2017;9(2):342-353.
